# Early Detection Intervals for Evaluating Event-Based Surveillance System: Reference Dataset Development Study

**DOI:** 10.2196/87030

**Published:** 2026-05-05

**Authors:** Yannan Shen, Philip AbdelMalik, Russell J Steele, David L Buckeridge

**Affiliations:** 1 Department of Epidemiology, Biostatistics and Occupational Health, School of Population and Global Health, Faculty of Medicine and Health Sciences, McGill University, Suite 1200, 2001 McGill College Avenue, Montreal, QC, H3A 1G1, Canada, 1 514-396-1153; 2 Department of Pandemic and Epidemic Intelligence Systems, World Health Organization, Berlin, Germany; 3 Department of Mathematics and Statistics, Faculty of Science, McGill University, Montreal, QC, Canada

**Keywords:** event-based surveillance, public health surveillance, surveillance, evaluation, infectious disease outbreak, dataset, data quality, detection, COVID-19, SARS-CoV-2

## Abstract

**Background:**

Early detection of health threats is an objective of public health surveillance, and event-based surveillance (EBS) using unstructured information from diverse sources has played an increasingly important role in achieving this objective. However, the evaluation of EBS systems has been hindered by the lack of reference data on outbreak onsets.

**Objective:**

We introduce the concept of an “early detection interval” and create a dataset of these intervals across multiple countries for the epidemic caused by the Omicron variant of SARS-CoV-2.

**Methods:**

We defined the early detection interval as the time between the date of introduction of an infectious agent to a country and the date at which an increase is detectable in traditional public health surveillance data. To determine the date of the introduction of the Omicron variant, we analyzed phylogenetic studies and genome databases. We estimated the end of the interval by applying Bayesian online change point detection to reported COVID-19 case counts. In addition to the early detection intervals, this dataset also contains variables indicating data quality. To further understand the variation in the lengths of the early detection intervals, stratified analysis and univariate Cox proportional hazards were implemented.

**Results:**

This dataset contains early detection intervals for the Omicron variant in 117 countries. The intervals have a median length of 28 (IQR 18-44) days, with a median beginning date of November 27, 2021 (IQR November 17, 2021, to December 12, 2021), and a median ending date of January 2, 2022 (IQR December 19, 2021, to January 9, 2022). Countries with high sequencing availability tend to have earlier start dates with a maximum difference across data sources of only 15 (IQR 7‐39) days and consequently a prolonged interval length with a median length of 29 (20-47) days. Countries with low incomes were underrepresented in this dataset, with only 12 (29.27%) out of 41 included, and they tended to have shorter intervals with a median duration of 16 (IQR 12‐23) days. The univariate Cox proportional hazards ratio regression analysis confirmed prolonged interval length in countries with high sequencing availability (hazard ratio 0.59, 95% CI 0.38‐0.92) and shortened interval length in low-income countries (hazard ratio 2.37, 95% CI 1.29‐4.36).

**Conclusions:**

The dataset of early detection intervals created in this study can serve as reference data and facilitate the evaluation of the timeliness of alerts generated by EBS systems. Systematic and comprehensive evaluation of EBS is important to guide the development of EBS and motivate the integration of EBS into public health practice. Our study also highlights cross-country disparities in data quality, particularly for genomic evidence, and the need for data collection and sharing focused on low-resource settings.

## Introduction

Infectious diseases continue to pose a major threat to global health [1,2]. As a recent example, the COVID-19 pandemic resulted in over 777 million cases and 7 million deaths reported as of May 2025 [3]. Early detection of infectious disease outbreaks is important for initiating timely prevention and mitigation measures to minimize health and social impacts [1,2,4]. To improve the timeliness and sensitivity of outbreak detection, public health agencies conduct surveillance using various information sources, such as online news and social media (known as event-based surveillance; EBS), as a complement to traditional case- and laboratory-based surveillance data (known as indicator-based surveillance; IBS) [5-7].

Evaluating the performance of EBS is crucial to ensure the objective of early detection is achieved, to understand the value of different surveillance methods, and to identify opportunities for performance improvement, but limited studies have comprehensively assessed EBS performance [4,8-12]. In particular, timeliness is one of the most important attributes for evaluating EBS systems aiming at the early detection of outbreaks [4,6,9,13]. Intrinsic timeliness measures the time from the start of an outbreak to an EBS alert [6,13]. Although the start of an outbreak is usually defined as “the earliest epidemiologically linked symptom onset or death,” the intrinsic timeliness of EBS alerts was found in several studies to range from 19 days to 3 months after the first retrospectively identified cases [14,15]. However, retrospective analyses of genomic data have revealed that the transmission of viruses among a population can occur weeks before the index case is retrospectively identified, which may provide a better approximation of the ground truth compared to the index case approach, for evaluating EBS timeliness [6,16-20]. For example, phylogenetic estimation has shown that the earliest transmission of the Omicron variant in South Africa occurred over a month before the reporting of the index case [18,21].

Extrinsic timeliness measures the time interval from EBS alerts to the notification or report of the same event from official sources [6,8,11,12,22-24]. In previous evaluation studies, the timeliness of EBS detection compared to official reporting ranged from −1 (ie, 1 day lagged behind) to 20 days, with results varying by disease, EBS system, and geographic region [8,11,23,24]. Official reports are generally well documented and relatively easy to access but are subject to reporting delays, which may lead to overestimating extrinsic timeliness. Meanwhile, the interpretation of extrinsic timeliness alone can be ambiguous, as alerts occurring much earlier than the official report may not be related to the corresponding outbreak. Additionally, a few studies use a fixed time window to define timely EBS detection. For example, Ganser et al [10] categorized EBS detections as timely if they occurred within a fixed, 4-week time window and found that only 9.2% of seasonal influenza outbreaks were detected in a timely manner by EBS systems. However, such a fixed time is arbitrary, even with some justification based on disease-specific knowledge, and, more importantly, ignores possible different contexts across countries, which may consequently lead to the misclassification of alerts.

In this study, we develop a dataset of intervals based on genomic evidence and IBS data that can serve as a reference framework for evaluating the timeliness of EBS, allowing the assessment of the potential contribution of EBS to early outbreak detection, prior to detection through IBS. We introduce the concept of an early detection interval, which is the time between the earliest evidence of pathogen introduction in a population and the earliest signals from routine analysis of traditional IBS data. Specifically, using the example of the Omicron variant, we build a dataset of early detection intervals that begin with the earliest introduction of the Omicron variant based on genomic evidence and end with the detected increase in reported COVID-19 case counts. We present a descriptive analysis of this dataset to summarize the temporal distribution of the start and end of the early detection intervals and explore the country-level factors associated with the length of the early detection interval using Cox proportional hazards regression models.

## Methods

### Study Design

In this study, we created a dataset of early detection intervals for the earliest subvariants of the Omicron variant of SARS-CoV-2 (ie, BA.1 and BA.2) in countries worldwide from the end of 2021 to the beginning of 2022. The early detection interval was defined as the time from the earliest genetic evidence of the Omicron variant’s introduction among the population in a country to the detectable increase in reported COVID-19 incidence cases.

**Figure 1.**
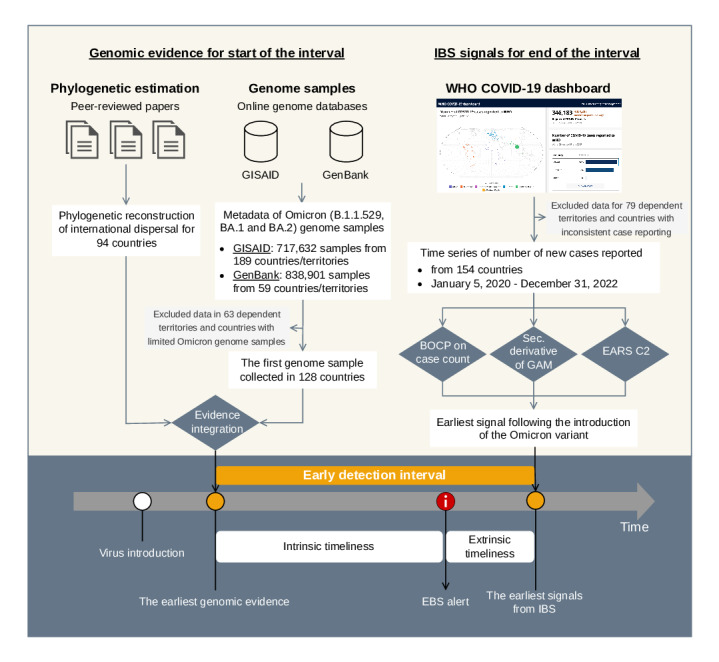
Illustration of the study design for creating the early detection intervals for the Omicron variant in countries worldwide, including the data sources, data inclusion and exclusion, and analytical methods used for generating the beginning and end of early detection intervals. BOCP: Bayesian online change point; EBS: event-based surveillance; EARS: Early Aberration Reporting Systems; GAM: generalized additive model; GISAID: Global Initiative on Sharing All Influenza Data; IBS: indicator-based surveillance.

To identify the beginning and end of the early detection interval in different countries, we conducted a retrospective analysis by compiling and linking genomic evidence from published papers and 2 online genome databases and detecting change points from COVID-19 incidence cases reported in different countries, as shown in Figure 1. In addition to the early detection interval, we also created supplementary variables to support users in assessing the data quality and potential biases of the early detection intervals.

We included countries with at least 1 data source for identifying the beginning of the interval and available case data. We excluded dependent territories from our study as our analysis was at the country level. The dependent territories tended to have different transmission patterns and capacities for public health surveillance and reporting compared to their sovereign states, while the available data sources had limited granularity to reflect within-country variation. To ensure the reliability of the interval, we also excluded countries with limited genome surveillance, data accessibility, and unreliable case reporting, as described in the following subsections.

### The Start of the Early Detection Intervals

The early detection interval in a country should begin when a pathogen (ie, the Omicron variant in this study) is first introduced to and begins circulating among the population. Among the multiple subvariants of the Omicron variant, we considered BA.1 and BA.2 only, as they are the earliest subvariants emerging at a similar time. Despite their genetic and epidemiological differences, both BA.1 and BA.2 have high transmissibility contributing to a new wave of COVID-19 cases in different countries. However, this ground truth of virus introduction and the start of transmission among the population is generally not possible to capture exactly, given the limited sample collection and sequencing capacity in most countries. Therefore, our definition of the start of an early detection interval was the earliest date of genomic evidence of the circulation of either the BA.1 or BA.2 Omicron variant in a country.

The primary data source for such genomic evidence is published studies estimating the initial introduction of the Omicron variant in different countries using phylogenetic reconstruction methods (Table S1 in Multimedia Appendix 1) [18,25-30]. One of the main studies we used was conducted by Tegally et al [25], which provides the reconstruction of the international dispersal patterns of variants of concern from Alpha to Omicron inferred from a representative sample of genome sequences using ancestral state reconstruction of discrete spatial locations for each variant of concern. From this study, we obtained global dispersal data, including the origin, destination, and date of international dispersal, estimated by 10 repeated phylogenetic analyses. We identified the earliest introduction of the Omicron variant in a destination country as the median of the 10 earliest international dispersals estimated. There are two countries, Bulgaria and Romania, where the median of the earliest exportation occurred a few days earlier than that of the earliest introduction. In this case, we modified the earliest introduction to the lower quartile of its estimation to ensure that the introduction occurred before exportation and to avoid confusion.

As many countries are not covered in the published studies identified, we also used the first collection date of Omicron genome samples to define the beginning of the interval. We collected the metadata of genome samples from 2 of the most popular genome databases that host large numbers of genome data on SARS-CoV-2 and have been widely used by researchers worldwide, the Global Initiative on Sharing All Influenza Data (GISAID) and GenBank hosted and maintained by the Federal Ministry of Food and Agriculture in Germany and the National Institutes of Health in the United States, respectively [31-33]. Both databases rely on the voluntary submission of genome data from laboratories worldwide with unknown sampling processes. Consequently, the two databases have different geographic coverage and focus despite a large degree of overlap. We tried to mitigate these biases to some extent by using these databases together so that we could identify the earliest possible collection of Omicron genome samples. Using the filter function of each platform, we selected Omicron genome samples collected after October 1, 2021, and obtained their metadata. Based on the date of sample collection in the metadata, we identified the first Omicron genome sample collected from human or environmental samples (eg, wastewater) in different countries.

For each country, we matched phylogenetic estimates from published studies with the earliest Omicron genome collection dates from GISAID and GenBank and then used the earliest of these as the beginning of early detection intervals. To indicate the quality of the data sources and the reliability of the genome evidence, we documented the number of data sources available for defining the beginning of an interval and computed the minimal and maximum temporal gap of the earliest evidence among these data sources. Meanwhile, for each genome database, countries were marked as having questionable data quality if a limited number of Omicron genome samples (ie, lower than 10% percentile in a database) or all samples were submitted on the same date, which might indicate a lack of sufficient and consistent sequencing and reporting efforts in these countries to provide reliable genome evidence. We excluded countries if they had evidence from only 1 genome database, and their data quality in this genome database was marked as questionable.

### The End of the Early Detection Intervals

The end of the early detection interval was defined as the earliest alert of a potential outbreak captured by traditional IBS. We assumed that alerts from other surveillance systems would add little value to early detection after this date. The number of new cases indexed by time and geolocation is one of the most commonly used indicators for early outbreak detection in IBS, as case counts are a timelier indicator than other metrics, such as the frequency of hospitalizations and deaths. We collected the weekly number of new COVID-19 cases reported from January 5, 2020, to December 31, 2022, from the World Health Organization (WHO) COVID-19 dashboard, which uses the date of reporting (rather than the date of symptom onset) for the WHO definition of a confirmed case of SARS-CoV-2 infection: “(a) a person with a positive Nucleic Acid Amplification Test, regardless of clinical criteria OR epidemiological criteria; (b) a person meeting clinical criteria AND/OR epidemiological criteria with a positive professional-use or self-test SARS-CoV-2 Antigen rapid diagnostic test” [3]. We excluded countries when the first case was reported later than May 31, 2020 (ie, no COVID-19 cases have been reported for nearly 3 months after the announcement of the global pandemic, which is unlikely to reflect transmission dynamics but reporting errors) or when no incident cases were reported in over one-third of the interval, both of which suggest inconsistent or delayed case identification and public health surveillance reporting.

We applied the Bayesian online change point (BOCP) detection model, the second derivative of the generalized additive model (GAM), and the Early Aberration Reporting Systems (EARS) C2 method to the entire time series of the number of new COVID-19 cases reported [34-40]. The change point detection methods have been applied to detect the beginning and end of outbreaks, and both models selected are reported to have good performance in some previous studies [10,35,36,41]. The BOCP algorithm assumed that the differenced case count could be divided into nonoverlapping partitions, and within each partition, it followed a Gaussian distribution. For countries with low case counts reported (ie, a median weekly count lower than 100), a BOCP model assuming a Poisson distribution was used. The differenced case count was used to remove the autocorrelation and stabilize the data. The BOCP analysis was implemented using the R package ocp (version 0.1.1). The change points were selected based on the global maximum of the posterior distribution of all change points. We also detected the change points of the growth rate of the case count using a GAM, assuming a negative binomial distribution with a log link. In the GAM, a single explanatory variable of time in weeks was included and modeled using thin plate splines. We also implemented a simple aberration detection method, that is, the EARS C2 method, as a baseline method for the BOCP and GAM models, using the R package surveillance (version 1.24.1) [38-40]. The EARS C2 method compares the observed case count with the most recent baseline, calculated as the moving average of the case count reported 3 to 5 weeks before the observed case count.

We identified the end of the early detection interval as the earliest change point detected by any of the models that occurred between the 7 days after the introduction of the Omicron variant (ie, the beginning of the interval) and the date of the maximum number of case counts reported within the subsequent 120 days. By doing so, we enforced a minimal interval length of 7 days, which is approximately 2 incubation periods of Omicron. We also recorded (1) the number of change point detection models that agreed on the selected point and (2) the temporal gap from the selected point to the following peak of case counts (Table 1). These measures provide future users with information about the reliability of the estimated interval end.

**Table 1.** The definitions of variables in the dataset of early detection intervals, including the beginning and end of the early intervals and data quality variables.

Variable	Working definition
Define the interval	
Country	Country where the transmission activity of the Omicron variant was taking place.
Date when the interval starts	The date when the earliest genomic evidence that indicates the dissemination of the Omicron variant among the population was presented, considering data from phylogenetic estimation and the earliest genome samples collected and submitted to GISAID^a^ and GenBank.
Date interval end	The date when the earliest increasing change point was detected from the case count time series by the highest number of models during the time between the 7 days after the beginning of the interval and the following peak of incidence case count reported within 120 days.
Length of interval	The number of days from the beginning to the end of an early detection interval.
Data quality for interval start	
Data source for interval start	In a country, the data source, among phylogenetic estimation, GISAID, and GenBank, that provides the earliest genomic evidence.
Number of data sources available for identifying the start of interval	In a country, the number of data sources, among the phylogenetic estimation, GISAID, or GenBank, that are available.
The minimal temporal gap across different data sources	In a country, if more than 2 data sources are available, the minimum difference in the time when the earliest evidence of transmission of the Omicron variant across data sources.
The maximum temporal gap across different data sources	In a country, if more than 2 data sources are available, the maximum difference in the time when the earliest evidence of transmission of the Omicron variant across data sources.
Data quality for interval end	
Completeness score	In a country, the proportion of weeks with new cases reported in the case count dataset.
Number of change point detection models that agree on the selected end point	In a country, the number of the change point detection models, including BOCP^b^ on differenced count, second derivative of GAM^c^ and EARS^d^ C2, detected the selected end point.
Temporal gap from the interval end to the following peak of case count	For a country, the number of days from the end of the early detection interval to the following peak of incidence case count reported within 120 days.

a GISAID: Global Initiative on Sharing All Influenza Data.

b BOCP: Bayesian online change point.

c GAM: generalized additive model.

d EARS: Early Aberration Reporting Systems.

### Statistical Analysis

We computed the median and IQR of the timing of the start and end of the interval and length of the early detection intervals for all included countries and by strata of countries based on the WHO regions and income levels (ie, low-income countries [LICs], lower-middle-income countries, upper-middle-income countries, and high-income countries [HICs]), based on the World Bank’s classification of the economies and public availability of genomic sequencing of countries (ie, low, moderate, and high availability based on a previous study) [42]. For levels of sequencing availability, we merged countries with low and moderate availability into 1 group to ensure sufficient observations for stable and reliable estimates. We compared the median of the length of intervals in different strata using the Wilcoxon rank-sum test (for 2-level strata) and the Kruskal-Wallis test (for multilevel strata). We also used univariate Cox proportional hazards regression models to assess factors associated with the hazard of the end of the interval. With the interval length as the outcome, the factors explored included income level (LICs vs non-LICs), sequencing availability, and indicators of data quality for genomic evidence, such as the number of data sources available and the maximum difference in the timing for the earliest genomic evidence across data sources. We verified if the proportional hazards assumption held by testing the correlation of scaled Schoenfeld residuals with time.

### Ethical Considerations

The original data in this study were aggregated and nonidentified, which are publicly accessible from the WHO COVID-19 dashboard [3], GenBank [33], and GISAID [31,32]. Thus, ethical approval and participant consent were not required for this study.

## Results

### Interval Characteristics

In this study, we collected phylogenetic estimates of viral introduction for 94 countries; metadata of 717,632 and 838,901 Omicron variant genome samples collected in 189 and 59 countries and territories from GISAID and GenBank, respectively; and case count data for 233 countries and territories from the WHO COVID-19 dashboard. After applying the inclusion and exclusion criteria, 117 countries were included in our study, with 46 out of 70 (65.71%) HICs and 12 out of 41 (29.27%) LICs included. The early detection intervals began on a median date of November 27, 2021 (IQR November 17, 2021, to December 12, 2021) and ended on a median date of January 2, 2022 (IQR December 19, 2021, to January 9, 2022), with a median length of 28 (IQR 18-44) days (Table 2).

**Table 2.** Distribution of the intervals for the early detection of the Omicron variant and data quality variables for 117 countries included and different groups of countries.

	Number of countries	Beginning of interval, median (IQR)	End of interval, median (IQR)	Interval length (days), median (IQR)	Max difference across data sources (days), median (IQR)	Gap to the peak of case count (days), median (IQR)
Total	117	November 27, 2021 (November 17, 2021, to December 12, 2021)	January 2, 2022 (December 19, 2021, to January 9, 2022)	28 (18-44)	16 (8-41)	28 (14-42)
Sequencing availability						
High	83	November 22, 2021 (November 16, 2021, to December 6, 2021)	December 26, 2021 (December 15, 2021, to January 9, 2022)	29 (20-47)	15 (7-39)	21 (14-28)
Low to moderate	27	December 12, 2021 (November 26, 2021, to December 16, 2021)	January 2, 2022 (December 12, 2021, to January 9, 2022)	23 (13-34)	27 (16-50)	28 (21-49)
Country income group						
LICs^a^	12	November 29, 2021 (November 23, 2021, to December 14, 2021)	December 19, 2021 (December 10, 2021, to December 27, 2021)	16 (12-23)	23 (17-43)	14 (12-16)
LMICs^b^	25	November 30, 2021 (November 13, 2021, to December 17, 2021)	January 2, 2022 (December 12, 2021, to January 16, 2022)	30 (23-43)	22 (13-54)	21 (14-28)
UMICs^c^	34	November 27, 2021 (November 19, 2021, to December 12, 2021)	January 2, 2022 (December 26, 2021, to January 9, 2022)	33 (21-47)	29 (13-58)	28 (21-35)
HICs^d^	46	November 22, 2021 (November 16, 2021, to December 7, 2021)	December 26, 2021 (December 5, 2021, to January 2, 2022)	27 (18-41)	8 (4-26)	35 (21-54)
WHO regions						
African Region	21	November 21, 2021 (October 14, 2021, to November 30, 2021)	December 12, 2021 (December 5, 2021, to December 19, 2021)	21 (12-47)	33 (16-59)	14 (14-28)
Region of the Americas	19	December 2, 2021 (November 20, 2021, to December 16, 2021)	January 2, 2022 (December 22, 2021, to January 5, 2022)	27 (19-37)	16 (11-30)	28 (18-32)
Eastern Mediterranean Region	16	December 1, 2021 (November 25, 2021, to December 9, 2021)	December 26, 2021 (December 19, 2021, to January 10, 2022)	19 (11-31)	26 (5-46)	25 (19-42)
European Region	42	November 22, 2021 (November 15, 2021, to December 12, 2021)	January 2, 2022 (December 19, 2021, to January 9, 2022)	28 (18-45)	11 (5-26)	28 (21-47)
South-East Asia Region	7	November 27, 2021 (November 24, 2021, to November 28, 2021)	January 2, 2022 (January 2, 2022, to January 12, 2022)	44 (36-46)	26 (8-42)	28 (25-46)
Western Pacific Region	12	December 4, 2021 (November 19, 2021, to December 17, 2021)	January 16, 2022 (January 2, 2022, to January 23, 2022)	37 (29-52)	32 (12-57)	35 (19-53)

a LIC: low-income country.

b LMIC: lower-middle-income country.

c UMIC: upper-middle-income country.

d HIC: high-income country.

When defining the beginning of an interval, 33 out of 117 (28.21%) countries were based on the phylogenetic estimation, whereas 84 out of 117 (71.79%) countries were based on the date of the first collection of the Omicron genome sample, mainly from GISAID. The phylogenetic estimations were, on average, 7 (IQR 4‐11) days earlier than the first collection in GISAID for 35 countries and 24 (IQR 14‐39) days earlier than the first collection in GenBank for 20 countries. Among 100 out of 117 (85.47%) countries with more than 1 data source to define the beginning of an interval, the maximum difference across different data sources was a median of 16 (IQR 8‐41) days, and the minimum value for this difference was a median of 13 (IQR 4‐32) days. Meanwhile, when defining the end of an interval, the completeness score of the COVID-19 case count data for the included countries had a median of 94.23% (IQR 92.31%‐94.87%). Most countries (101/117, 86.32%) had the end of their interval detected by either the BOCP model or the GAM model, and in only 16 out of 117 (13.68%) countries, the EARS C2 method detected an increase in case counts prior to detection by the BOCP or GAM models. The number of new COVID-19 cases reported peaked after a median of 18 (IQR 14‐42) days after the end of the interval.

### Factors Associated with the Length of the Early Detection Interval

In a stratified analysis, countries with high sequencing availability had the smallest range in earliest transmission evidence across 3 data sources and a lead of 12 (95% CI 5‐21) days at the beginning of the interval, compared with countries with moderate or low sequencing availability (Table S2 and Figure S1 in MultimediaAppendices 2  3, respectively). Conversely, the intervals began on similar dates across countries with various income levels and ended 7 (95% CI −0.00004 to 21) days earlier in LICs compared to non-LICs, resulting in intervals that were a median of 11 (95% CI 3‐20) days shorter. However, the case count peaked about 2 weeks after the end of intervals in LICs, which was 14 (95% CI 7‐21) days shorter than in non-LICs. Meanwhile, in HICs, the case count peaked at a median of 35 (IQR 21‐54) days after the end of intervals. HICs also had consistent genomic evidence for the introduction of the Omicron variant, with the maximum gap across different sources of 8 (IQR 4‐26) days, which was 12 (95% CI 5‐20) days shorter than that of other countries. Across different WHO regions, the earliest signals from case counts occurred first in the African Region on December 19, 2021 (IQR December 10, 2021 to December 27, 2021), which was about 2 weeks before the peak of new COVID-19 cases occurred, while this signal was the latest in the Western Pacific Region, which was 1 month after the same signal in the African Region and 5 weeks before the peak of COVID-19 cases in the Western Pacific Region.

The univariate Cox proportional hazards ratio regression analysis confirmed prolonged interval length in countries with high sequencing availability, with a hazard ratio of 0.59 (95% CI 0.38‐0.92), and shortened interval length in LICs, with a hazard ratio of 2.37 (95% CI 1.29‐4.36; Table 3). The other 2 variables, the number of data sources available and the maximum difference across different data sources for defining the beginning of intervals, were significantly associated with prolonged intervals with an estimated hazard ratio below 1, both of which indicated the reliability of the data quality of the beginning of intervals.

**Table 3.** The estimated hazard ratio and its 95% CI from univariate Cox proportional hazards regression analysis of the lengths of the early detection interval in 117 countries included.

Predictor	Number of events	Hazard ratio (95% CI)	*P* value
Maximum difference across data sources for interval start	100	0.98 (0.97-0.99)	.004
Number of data sources available for interval start	117	0.65 (0.48, 0.88)	.005
Country income group			
Non-LICs^a^	105	Reference	
LICs	12	2.37 (1.29-4.36)	.006
Sequencing availability			
Low to moderate	27	Reference	
High	83	0.59 (0.38-0.92)	.02

a LIC: low-income country.

## Discussion

### Principal Findings

In this study, we defined an early detection interval as the time between the date of the earliest genomic evidence of the presence of a pathogen in a country and the date of the first alert from an IBS system, which is conceptually tailored to support evaluating early outbreak alerts with well-defined boundaries. We created a dataset of such intervals for the early detection of the Omicron variant in 117 countries worldwide with a median start date of November 27, 2021 (IQR November 17, 2021, to December 12, 2021) and an end date of January 2, 2022 (IQR December 19, 2021, to January 9, 2022) [43]. We also observed interval lengths ranging from 7 to 66 days across countries, which may reflect the influence of factors such as the capacity for sequencing and data sharing in genomic surveillance, the capacity for case identification and reporting of IBS systems, and the transmission dynamics of the Omicron variant. This dataset can serve as a reference dataset in future evaluation studies to evaluate both intrinsic and extrinsic timeliness of EBS.

The early detection interval begins, in theory, with the initial introduction of the Omicron variant into a population, as only then can the variant cause excess infections and be detected in the population. Yet, the timing of introduction is difficult to measure consistently across countries with existing data, so we approximated this date using the earliest genomic evidence of the presence of the variant, retrospectively identified. Retrospective analysis of genomic data has been widely adopted to understand transmission dynamics and the impact of virus variants, and it can generate evidence uncovering the transmission of specific substrains of viruses such as SARS-CoV-2 among a population, in some cases weeks or even months before other data sources [16-20]. High-quality genomic evidence that closely approximates the introduction of the Omicron variant allows capturing the contribution of early EBS alerts. When used as a reference dataset in evaluation studies, this early detection interval can avoid the underestimation of the intrinsic timeliness of EBS alerts compared to previous studies that have used the first retrospectively identified case to approximate the beginning of an outbreak [14,15].

We found that countries with high sequencing availability and income levels tended to have concordant genomic evidence across data sources (ie, an indicator of the data quality of genomic evidence we compiled) and therefore a relatively earlier start of the intervals than those of other countries. Genomic data may have uneven quality across countries due to differences in sequencing capacities, sampling strategies, and data reporting and sharing, which consequently translate into biases in the measurement of the start of intervals [16,42]. We integrated several data sources to identify the earliest possible genomic evidence for the introduction and transmission of the Omicron variant. We found that integrating multiple sources for genomic evidence may help reduce disparities in early detection, compared to using a single database. For example, the phylogenetic estimations were earlier than the first collection in GISAID in 35 countries, with a median of 7 (IQR 4‐11) days of lead time. Specifically, South Africa had its retrospectively identified first collection of the Omicron genome sample 28 days before the index case reported [21], yet 11 days after the phylogenetic estimation of the earliest introduction.

The early detection interval ended with the detection of the earliest alerts from IBS data. Using this end of early detection intervals as a reference, we can assess the extrinsic timeliness that highlights the added value of EBS in comparison with the IBS alerts and the potential for accelerated response. Unlike several studies that compared EBS alerts with the first official reporting [6,8,11,44], this measurement also avoids overestimating the contribution of EBS alerts that occurred after the earliest IBS alerts. Using 3 different methods to detect signals from IBS data, we found that the EARS C2 method was likely to generate detections later than BOCP and GAM and missed the gradual and subtle increases, which aligns with findings in previous studies showing EARS methods to be inferior to regression-based methods for aberration detection [45,46]. We also found that the temporal distribution of the end of intervals mainly varied across countries in different geographic regions, aligning with the global dispersal pattern of the Omicron variant [25]. The reported case increase, however, may lag behind the increase in actual infections due to asymptomatic cases. Such a lag may be more evident in countries that were first impacted by the Omicron variant due to a lack of awareness before official identification [47,48]. A delayed end may cause the overestimation of the interval length and, when combined with a delayed start, shift the early detection interval later in time, both contributing to an overestimation of EBS’s extrinsic timeliness.

As for the length of early detection intervals, while it is not a measurement of the timeliness of IBS, the median length of approximately 1 month indicates the potential for improvement in early outbreak detection of a newly emerged variant of SARS-CoV-2. Our findings also demonstrate the substantial variation in the lengths of early detection intervals across countries. Our analysis mainly revealed the association between varying lengths and genomic data quality. Given this variation in data quality, the interval lengths should be interpreted with caution, considering potential biases. For example, shorter interval lengths observed in LICs compared to those in non-LICs in our study may indicate extensive underestimation of the introduction of the Omicron variant due to limited genomic surveillance, rather than a lower value of adopting EBS.

### Limitations

This study is subject to several limitations. First, the countries included in this dataset are not a representative sample. This dataset includes 117 countries, yet is overly represented by HICs, with 46 out of 70 (65.7%) HICs included. Conversely, less than half of the LICs (12/31, 38.7% countries) and countries in the African Region (21/51, 41.2% countries), the Region of the United States (19/54, 35.2% countries), and the Western Pacific Region (12/41, 29.3% countries) were included due to limited data availability. Therefore, although EBS generated timely signals in LIC included in our study, caution is warranted in generalizing our findings to all LICs. There is also a need for additional research to generate the data that would allow this reference dataset to be expanded to a larger and more representative sample of LICs.

Second, as we discussed earlier in this section, our measurements of both the start and end of the intervals are likely to be biased toward later in time, which can further bias the lengths of early detection intervals and the Cox analysis of the interval lengths. To minimize such biases, we designed our inclusion and exclusion criteria and compiled genomic evidence from several data sources. We also included several supporting variables alongside the early detection interval to support potential users in assessing the data quality and corresponding biases. However, the data quality variables may not fully reveal the potential biases, and we could not include additional quality metrics due to the limited metadata available for some sources. For example, the data collection and sampling processes and representativeness of both genomic data from GISAID and GenBank and reported incidence cases are unknown but very likely to be different across countries and be uneven within a country [16,49]. Moreover, although the models selected in our study are reported to have good performance in some previous studies [10,35,36,41], further evaluation using simulation data could help us to understand the performance of these models and identify important factors that could impact the accuracy of the measurement of early detection intervals. Therefore, the timeliness metrics measured using these early detection intervals should be interpreted in the context of each country and carefully considering measured and unmeasured biases.

Third, in terms of generalizability, the concept of the early detection interval can be applied to other infectious diseases for evaluating the timeliness of detection. However, it requires corresponding data of relatively high quality to be available. As the early detection intervals are designed to evaluate the timeliness of EBS, a substantial delay in case identification that is hard to resolve by improving practice within IBS will likely increase the value of EBS for early outbreak detection. Therefore, this early detection interval may also apply to some vector-borne diseases, such as dengue virus disease and Zika virus disease, as well as avian influenza. Early indication of outbreaks of these diseases can be observed from environmental, zoonotic, entomologic, and genomic data, while asymptomatic infections make early detection through IBS challenging. When generalizing this early detection interval to other contexts, the definition and measurement of the interval should be adapted to the study context, which in turn would alter the interpretation of the evaluation results. Conversely, this approach might not work well for diseases such as Ebola because of the small number of cases and the relatively low delay in case identification due to severe and abrupt symptoms.

Finally, while the dataset of early detection intervals can enable the evaluation of the added value of EBS relative to the timeliness of outbreak detection, it would also be informative if timeliness metrics could consider other outbreak milestones, for example, verification of a signal and the initiation of interventions [9,50,51]. In our study, we were unable to include these milestones in our dataset due to limited data available across countries. To allow the extension of evaluation to these milestones, public health agencies should document, store, and publish the data on the verification of EBS and IBS signals and on the initiation of interventions. Routine and systematic capture of these data would provide reference data to expand evaluation to allow a more comprehensive and accurate quantification of the impacts of EBS in accelerating response [9,13,51,52]. It should also be noted that this definition of early detection interval may exclude legitimate early EBS signals (eg, reports of unusual clusters prior to sequencing confirmation), which could underestimate the full potential contribution of EBS. In addition to timeliness, other attributes, such as sensitivity, precision, usefulness, representativeness, and flexibility, are also important for comprehensively evaluating the early outbreak detection capacity of EBS [4,6,9]. For instance, the early detection interval focuses on the temporal adjacency of EBS alerts to the target event yet may not reflect their geographic coverage and representativeness. Further analysis is needed to measure these attributes in parallel using quantitative and qualitative analyses [6,9,11].

### Conclusion

Our study created a dataset of early detection intervals for the Omicron variant in countries worldwide by retrospectively compiling and analyzing data from multiple sources. Using this dataset of early detection intervals as the reference data enables future evaluation studies to assess both intrinsic and extrinsic timeliness of EBS. Our study also highlights cross-country disparities in data quality, particularly for genomic evidence and the need for data collection and sharing, focusing on low-resource settings. While many EBS systems have been developed and improved over the past few decades, the dearth of systematic and structured evaluation has limited the integration of EBS systems into public health surveillance practice. The early detection interval dataset presented in this study addresses important barriers of the lack of reference data and enables evaluation studies to generate valuable evidence on the added value of EBS compared to traditional IBS and the contribution of EBS systems with different designs. This evidence should guide the development of EBS and motivate acceptance and integration of EBS into public health practice, which is key to achieving the goal of timely outbreak detection and response and building collaborative public health surveillance.

## Supplementary material

10.2196/87030 Multimedia Appendix 1 List of literature reporting the phylogenetic estimations of the introduction of the Omicron variant in countries.

10.2196/87030 Multimedia Appendix 2 List of countries included in the dataset of early detection intervals for the Omicron variant.

10.2196/87030 Multimedia Appendix 3 Temporal distribution of the beginning and end of early detection intervals.

## Abbreviations

BOCP: Bayesian online change point
EARS: Early Aberration Reporting Systems
EBS: event-based surveillance
GAM: generalized additive model
GISAID: Global Initiative on Sharing All Influenza Data
HIC: high-income country
IBS: indicator-based surveillance
LIC: low-income country
WHO: World Health Organization
