# Supplementary material for: Early Detection Intervals for Evaluating Event-Based Surveillance System: Reference Dataset Development Study
Source: JMIR Public Health Surveill. 2026 May 5;12:e87030. doi: 10.2196/87030 (PMC13143157; doi:10.2196/87030)
Supplement: Multimedia Appendix 1 [file publichealth-v12-e87030-s001.docx]

**Multimedia Appendix 1**

Table S1 List of literature reporting the phylogenetic estimations of introduction of Omicron variant in countries

| Title | Authors | Year of Published | Countries |
| --- | --- | --- | --- |
| Dispersal patterns and influence of air travel during the global expansion of SARS-CoV-2 variants of concern | Tegally et al | 2023 | 94 countries |
| Rapid epidemic expansion of the SARS-CoV-2 Omicron variant in southern Africa | Viana et al | 2022 | South Africa |
| Genomic epidemiology of SARSCoV‑2 Omicron variants in the Republic of Korea | Lee et al | 2022 | Republic of Korea |
| Genomic assessment of invasion dynamics of SARS-CoV-2 Omicron BA.1. | Tsui et al. | 2023 | UK |
| Genome Evolution and Early Introductions of the SARS-CoV-2 Omicron Variant in Mexico | Castelán-Sánchez et al | 2022 | Mexico |
| Lineage BA.2 dominated the Omicron SARS-CoV-2 epidemic wave in the Philippines | Li et al | 2022 | Philippines |
| Genomic Epidemiology of the Main SARS-CoV-2 Variants Circulating in Italy During the Omicron Era | Bergna et al | 2025 | Italy |
