# Supplementary material for: Early Detection Intervals for Evaluating Event-Based Surveillance System: Reference Dataset Development Study
Source: JMIR Public Health Surveill. 2026 May 5;12:e87030. doi: 10.2196/87030 (PMC13143157; doi:10.2196/87030)
Supplement: Multimedia Appendix 3 [file publichealth-v12-e87030-s003.docx]

**Multimedia Appendix 3**

Figure S1 Temporal distribution of the beginning (red) and end (green) of early detection intervals across included countries. Top: Countries with high sequencing availability; Bottom: Countries with moderate or low sequencing availability
